# A novel heterogeneous network-based method for drug response prediction in cancer cell lines

**DOI:** 10.1038/s41598-018-21622-4

**Published:** 2018-02-20

**Authors:** Fei Zhang, Minghui Wang, Jianing Xi, Jianghong Yang, Ao Li

**Affiliations:** 1 School of Information Science and Technology, University of Science and Technology of China, Hefei, AH230027 China; 2 Centers for Biomedical Engineering, University of Science and Technology of China, Hefei, AH230027 China

## Abstract

An enduring challenge in personalized medicine lies in selecting a suitable drug for each individual patient. Here we concentrate on predicting drug responses based on a cohort of genomic, chemical structure, and target information. Therefore, a recently study such as GDSC has provided an unprecedented opportunity to infer the potential relationships between cell line and drug. While existing approach rely primarily on regression, classification or multiple kernel learning to predict drug responses. Synthetic approach indicates drug target and protein-protein interaction could have the potential to improve the prediction performance of drug response. In this study, we propose a novel heterogeneous network-based method, named as HNMDRP, to accurately predict cell line-drug associations through incorporating heterogeneity relationship among cell line, drug and target. Compared to previous study, HNMDRP can make good use of above heterogeneous information to predict drug responses. The validity of our method is verified not only by plotting the ROC curve, but also by predicting novel cell line-drug sensitive associations which have dependable literature evidences. This allows us possibly to suggest potential sensitive associations among cell lines and drugs. Matlab and R codes of HNMDRP can be found at following https://github.com/USTC-HIlab/HNMDRP.

## Introduction

Over the past 20 years, significant improvement in genomic profiling technologies have make it possible that personalized medicine become the fashion trend of future medical science^1,2^. In comparison with the paradigm of conventional symptoms-oriented drug discovery and development, personalized treatment makes use of tumor response and vulnerability to handle the expensive and limitations in clinical experiments. The major challenge in personalized prevention and treatment is the identification of biomarkers which is critical to understand the pathogenesis of given complex disease^3^. However, researchers are required to consider the time and cost effectiveness of predictive biomarker in human or animal models as it is not feasible to test the clinical efficacy and toxicity of large populations of cancer patients with hundreds of drugs. High-throughput drug screening technologies enable many studies to conduct large-scale experiments on human cancer cell lines. For instance, two recent consortiums, GDSC^4^ (Genomics of Drug Sensitivity in Cancer) and CCLE^5^ (Cancer Cell Line Encyclopedia) have analyzed around 1500 cancer cell lines and their genomic profiles against 280 drugs. Both of two studies provide genome-wide data of multiple type of cancer cell lines and drug sensitivity data of established anticancer drugs against these cell lines.

For improving understanding of disease and potential personalized medicine, one burgeoning field of interest is the problem of drug response prediction^6^. So far many prediction methods have been developed to facilitate and speed up drug discovery^7^ and repositioning process. For example, Gupta *et al*. use genomic feature based model to predict anticancer drug responses and have achieved good results based on above dataset^8^. Dong *et al*. propose a SVM classification model to accurately predict drug sensitivity according to gene expression profile in the CCLE dataset and have attained good performance for several drugs^9^. Meanwhile, Geeleher *et al*. apply ridge regression model and use the same dataset to predict drug response and also obtain equally good performance^10^. This kind of approach underlines the use of cell line’s genomic information in drug response prediction. In addition, many studies begin to pay their attention to the use of heterogeneity relationships among cell line genomic alteration, cell line-drug sensitivity and drug chemical structure. For instance, Liu *et al*. develop a systematic algorithm to predict the anti-cancer drug response via combining both cell line genomic and compound structure features^11,12^. Menden *et al*. propose a machine learning model to accurately predict cell line-drug sensitivities using both the cell line’s genomic features and the drug’s chemical structure properties^13^. And Ammad-Ud-Din *et al*. propose a kernelized Bayesian matrix factorization method (KBMF) to predict drug response by integrating the same dataset of cell line genomic and drug chemical properties^14^. Based on the same principle, Wang *et al*. propose a kernel function to correlate the heterogeneous pharmacogenomics information of both cell and drug, and then use SVM classifier to infer the cell line-drug associations^15^. And Zhang *et al*. construct a dual-layer network between cell line and drug and use weighted model to efficiently predict anti-cancer drug response through incorporating similarity between cell line and drug^16^.

Despite aforementioned great works have achieved promising results, other factors contributing to predict cell line-drug associations lies in the fact drug-target and protein-protein interaction (PPI) information are often cooperated in drug discovery, which have been demonstrated in previous studies^17–20^. Recently, Stanfield *et al*. construct a heterogeneous network to compute network profiles for cell lines and drugs, then perform a random walk with restart to predict links between cell lines and drugs based on these profiles^21^. The authors show integrating cell line mutation data, drug responses with PPI network can significantly improve its prediction performance. Despite its effectiveness, drug-target interactions are not integrated into the heterogeneous network to compute network profiles and therefore may influence the prediction results.

Inspired by the above method, there is a strong incentive to combine genomic and compound information with drug-target and PPI interaction information to predict drug responses. Accordingly, we present a novel heterogeneous network-based method for drug response prediction, named HNMDRP, to efficiently predict cell line-drug associations by incorporating cell line genomic profile, drug chemical structure, drug-target and PPI information. We first introduce the similarity measure to construct this heterogeneous network model^22^ by calculating Pearson correlation coefficient between cell line genomic profiles, drug chemical structures and target gene. Subsequently, we perform an information flow-based algorithm^23^ on this network and obtain the score of all cell line-drug pair, where the score is the prediction of drug response. In order to validate the effectiveness of drug-target and PPI information in our cell line-drug-target heterogeneous network, we compare it with existing methods. To perform a proper evaluation on our novel heterogeneous network-based method, we implement leave-one-out cross validation (LOOCV) to demonstrate its superior performance compared with existing state-of-the-art methods: Zhang’s method^16^, Stanfield’s method^21^, DLNDRP^24^, SVMDRP. The comprehensive results show that our method achieves the best AUC values for most drugs. Besides, our method can retrieve the largest true cell line-drug sensitive associations when focusing on the top percent predicted cell-drug associations. We then use our HNMDRP method to find several novel potential sensitive associations according to high-ranking prediction results which are strongly supported by related literatures. These results provide convincing evidence of the good performance of HNMDRP as well as potential value in future biological experiments.

## Results

### Evaluation of prediction performance of HNMDRP

In this work, leave-one-out cross validation^25^ (LOOCV) is applied to evaluate the predictive performance of our HNMDRP method in predicting drug response between cell line and drug. At each step of LOOCV experiment^26^, consistent with previous studies^26–28^, we treat a sensitive association between a cell line and a drug as testing data by setting the value as 0 in the matrix *A*_*cd*_. The rest of all associations are treated as training data for model learning. But only the prediction score of testing data is extracted each time. This process is repeated until every sensitive association between cell line and drug is treated as testing data once. Actually, for each given drug, only those cell lines with known associations are ranked in descending order according to the prediction score of LOOCV experiment. Afterward, the receiver operating characteristic (ROC) curve is employed to show the predictive performance of our HNMDRP method and other methods by plotting true positive (sensitive) and false positive (resistant) at different cutoff points^22^. Here, true positive rate (TPR) represents the percentage of sensitive cases correctly labeled as positives, and false positive refers to the ratio of resistant cases incorrectly labeled as positive. At the same time, we also compare the predictive performance of our method when only removing each information that include drug’s 1-D and 2-D structure information, PPI information, gene-gene correlation information and target similarity network information. The experimental results (as shown in supplementary Figure S4) show that all information are vital for drug response prediction, and PPI and gene-gene correlation information play relatively more important role than others. In addition, The computational complexity is mainly determined by equations (4) and (5) and are O(*nm*^5^*l*^4^) and O(*n*^3^*m*^5^*l*^2^), respectively. Considering the fact that the number of cell lines(*n*) and number of drugs(*m*) are relatively smaller than number of target genes(*l*), thus, the main contribution of computational complexity is the target gene nodes(*l*). Accordingly, the overall complexity of our model is O(*n*^3^*m*^5^*l*^2^).

**Figure 1:**
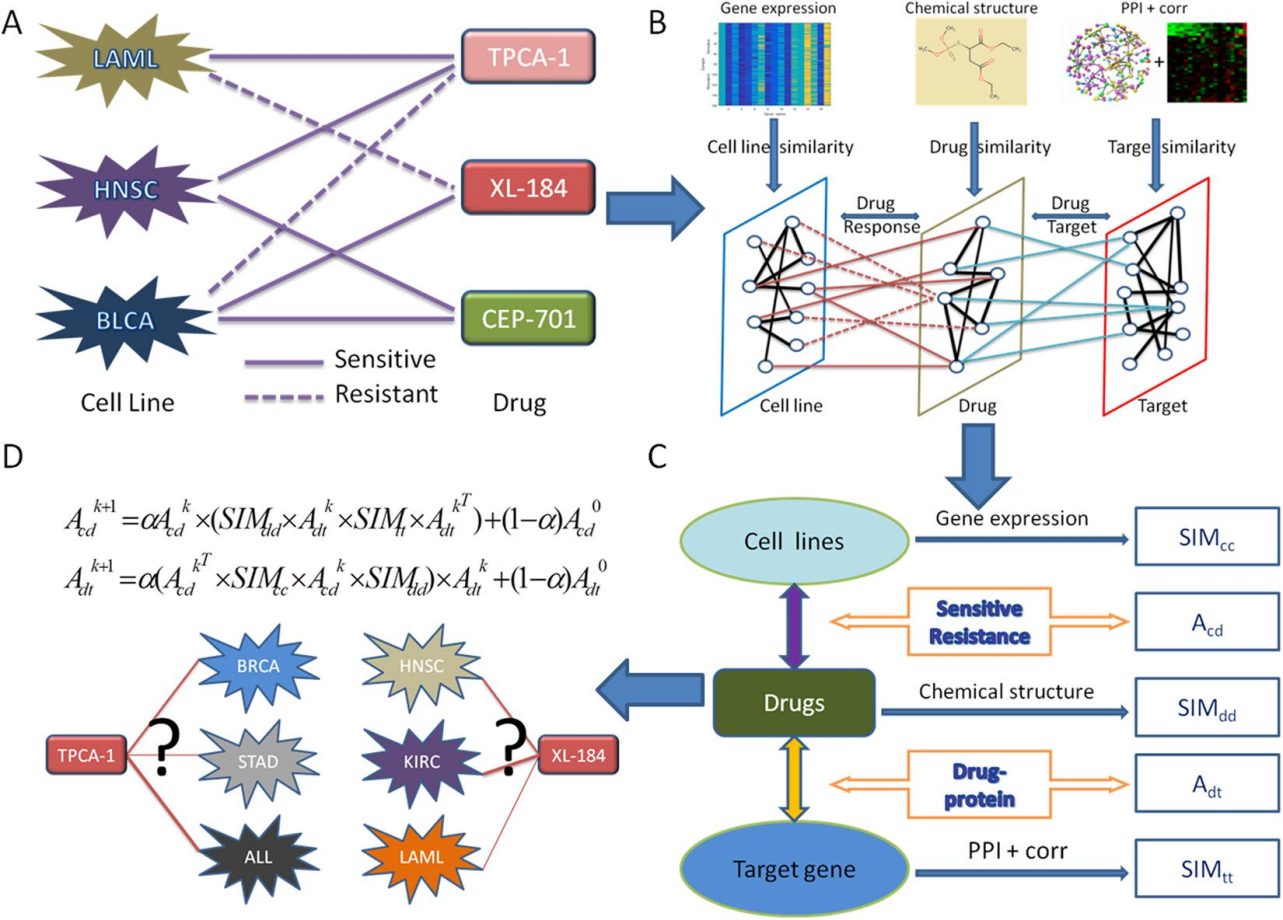
The overall workflow of our HNMDRP method. (**A**) Collecting known sensitive or resistant associations between cell lines and drugs. (**B**) Integrating heterogeneous information which includes cell line gene expression profile, drug chemical structure, drug-target and PPIs. (**C**) The schematic of our network model. Each sub-network is obtained to construct a comprehensive heterogeneous network. (**D**) Performing an information flow-based algorithm on the heterogeneous network.

### Compared with existing methods

**Figure 2:**
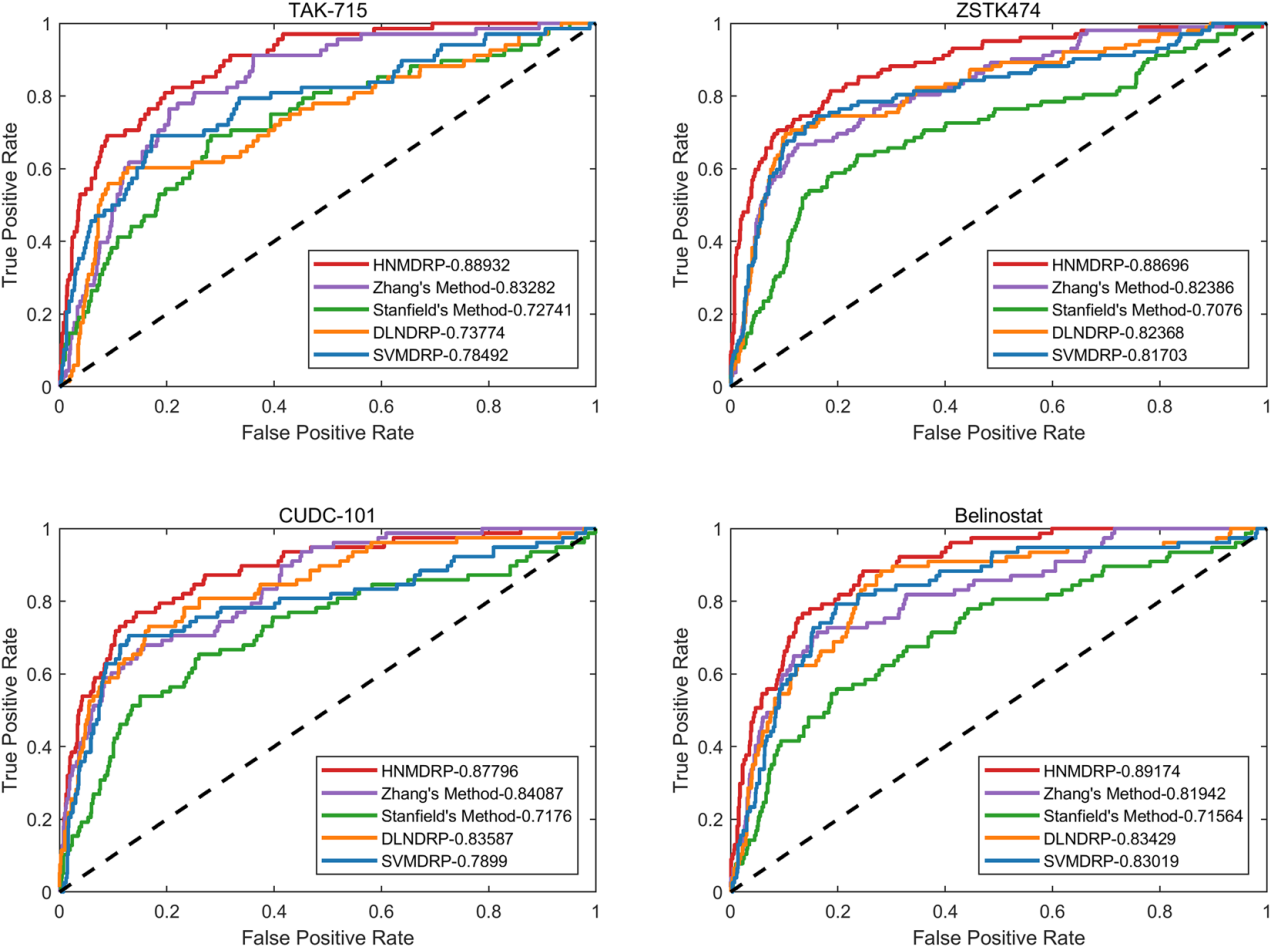
The ROC curve of drugs. Performance comparison of ROC curve among HNMDRP, Zhang’s method, Stanfield’s method, DLNDRP and SVMDRP method based on LOOCV.

In order to comprehensively assess the efficiency of our method on predicting drug responses, we compare HNMDRP method with state-of-the-art method: Zhang’s method, Stanfield’s method, DLNDRP and SVMDRP. Here, Zhang *et al*. propose a computational framework for the dual-layer integrated cell line-drug network to accurately predict tumor drug responses. And Stanfield’s method is performed on network profile which is computed by a large heterogeneous network to accurate and reproducible classification of drug sensitive and resistance. DLNDRP is a heterogeneous graph based inference on a two-layer network which consist of only cell line nodes and drug nodes for drug response prediction. SVMDRP is implemented on cell line gene expression and drug sensitivity data for predicting drug response. We made comparison of these five methods as shown in Fig. 2 and Table 1. From the results of Fig. 2, we find that our method achieve better results than both Stanfield’s method and Zhang’s method. In addition, as shown in Table 1, we can see that the average AUC value of our HNMDRP method are 5.6% and 14.26% higher than DLNDRP and SVMDRP, respectively. The results of remaining drugs are listed in Supplementary Table S3. The highest AUC value of 93.8% is obtained by drug SNX-2112 which also achieved good results using liquid chromatography method^27^. According to these results, we know that our method HNMDRP can predict drug responses more accurately than other state-of-the-art methods investigated here.

**Table 1:** The results of leave-one-out cross validation: AUC value of several drugs.

Drug	Method	AUC
SNX2112	HNMDRP	0.9380
	Zhang’s Method	0.9079
	Stanfield’s Method	0.7523
	DLNDRP	0.8896
	SVMDRP	0.8938
CAY10603	HNMDRP	0.9341
	Zhang’s Method	0.9103
	Stanfield’s Method	0.7733
	DLNDRP	0.8708
	SVMDRP	0.8692
CP466722	HNMDRP	0.9143
	Zhang’s Method	0.8669
	Stanfield’s Method	0.7787
	DLNDRP	0.8581
	SVMDRP	0.5955

### Tissue specific of cell line type

**Figure 3:**
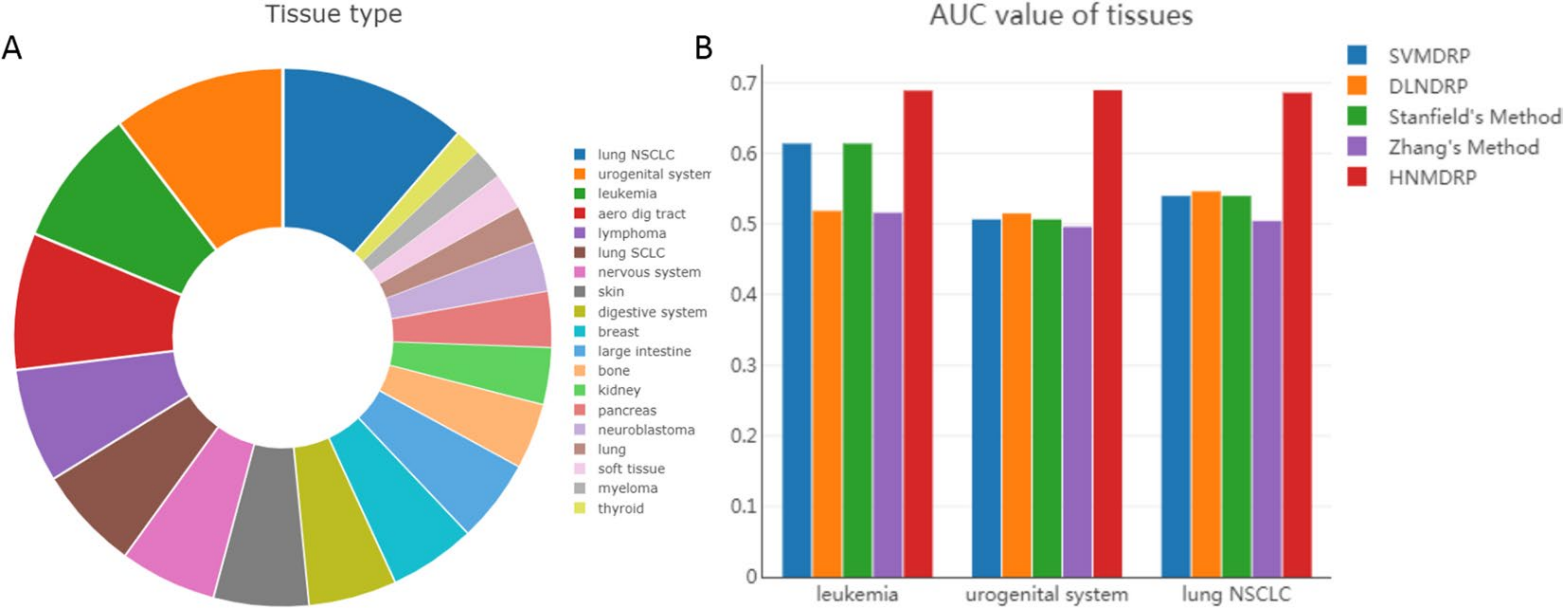
The performance of HNMDRP in diverse tissue types. (**A**) The distribution of each tissue types, including Lung, leukemia, breast, kidney and so on. (**B**) The AUC values of three major tissue types (leukemia, Lung NSCLC, urogenital system).

Drug responses may have large differences in diverse tissues types. Therefore, we test whether our HNMDRP can achieve a good performance when considering different cell line tissue types. As shown in Fig. 3A, 19 tissue types of cancer cell line and the distribution of these types are obtained based on GDSC dataset. We find that the major tissue types are leukemia (acute myeloid leukemia and chronic lymphocytic leukemia), urogenital system (bladder cancer), Lung NSCLC (non-small cell lung carcinoma). They take up 8.3% (80), 10.4% (100), 11.3% (109) on all 962 cancer cell lines, respectively. In order to demonstrate the comparable predictive results of our proposed method in different tissue types, we examine the performance on predicting drug responses in above three types of tissue. As shown in Fig. 3B, the bar represents the area under the ROC curve for three tissue types. And the average AUC values are 0.6787, 0.5053, 0.5534, 0.5265 and 0.5324 for five methods HNMDRP, Zhang’s method, Stanfield’s method, DLNDRP and SVMDRP on leukemia, urogenital system and lung NSCLC. These results indicate that our HNMDRP method can also achieve consistent performance on diverse tissue types. And the AUC values of the rest tissue types are listed in Supplementary Table S4. Furthermore, we only use the specific type of cell line to train our model and predict the drug responses based on these tissue types. The experimental results show that our method also achieve the best performance as shown in supplementary Figure S4.

### Case studies

**Figure 4:**
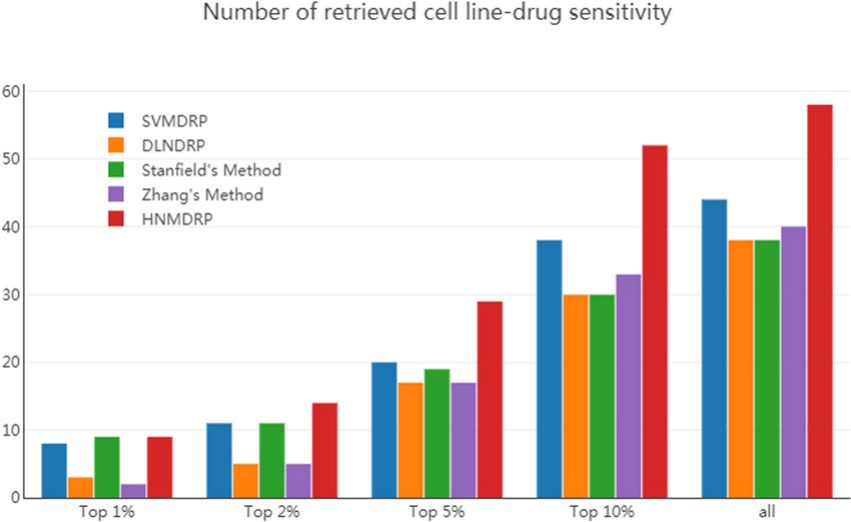
The number of correctly retrieved cell line-drug associations at different percentiles among five methods for drug GSK2126458.

It is known that the prediction results of false positive are usually suspicious in study of bioinformatics^28^. In this work, our HNMDRP method has attained a good performance in predicting known cell line-drug associations when compared with other existing method. We need to validate the ability of retrieving true positive (sensitive) associations in the prediction results among five methods. Thus, in addition to the ROC curves, we also compare the numbers of correctly retrieved cell line-drug sensitive associations according to different percentiles^29^. As shown in Fig. 4, we take drug GSK2126458 as an example, which have 94 positives (sensitive) and 808 negatives (resistant) associations, for each percentile p% (1%, 2%, 5%, 10% and 100%), we count the number of retrieved true positives among 962 cell lines based on the prediction results. And we can easily find that our HNMDRP method has little true positive predictions at percentiles 1% and 2%, but has significant more predictions at higher percentiles. These results indicate that HNMDRP method gives most of the known cell line-drug sensitive associations higher ranks and gives several unknown associations very high ranks.

Computationally predicted results usually need experimental verification, but it has more difficulty and limitation in practical implementation. Thus, similar to Wang *et al*.^15^, which find out novel sensitive associations based on the prediction score of cell line-drug pair with unknown associations in the database. To further test the ability of our HNMDRP method in predicting potential cell line-drug associations, we searched the top20 ranked candidate prediction results of all cell line-drug pair which have unknown association with drugs in GDSC dataset. As shown in Table 2, we find literature evidences to support those cell line-drug pairs be novel potential sensitive associations. For instance, the cell type of cell line MHH-CALL-2 is B cell leukemia, and the literature evidence provided by Lucas *et al*. indicate that the drug MS-275 is the promising treatment programs on this cancer cell line which is ranked 4 in prediction results^30^. Meanwhile, Gobin, *et al*. suggest that drug NVP-BEZ235 is the potential therapeutic strategy on cell line CHSA0011 of cell type chondrosarcoma, which is ranked 10 among all cell lines^31^. For drug Belinostat and cell line AMO-1, the published work^32^ gives evidence to clarify them be potential treatment in clinical trials. The remaining novel sensitive prediction results and literature evidences shown in Table 2 indicate that our HNMDRP method can accurately uncover novel sensitive associations between cancer cell line and drug, which provide a foundation of future experimental verification. Based on the above results, we can generally confirm that drug-target and PPI information are really important for drug response prediction.

**Table 2:** The top20 predictions of cell line-drug pairs (unknown) computed by HNMDRP which have literature evidences be novel sensitive associations.

Drug	Cell	Cell type	Drug usage	Rank
MS-275	MHH-CALL-2	B_cell_leukemia	B_cell_leukemia^30^	4
NVP-BEZ235	CHSA0011	Chondrosarcoma	Chondrosarcoma^31^	10
Belinostat	AMO-1	Haematopoietic_neoplasm	Myeloma^32^	12
VX-680	ML-2	Acute_myeloid_leukaemia	Myeloma^53^	17
Vorinostat	CCF-STTG1	Glioma	Glioma^54^	19
Roscovitine	MKN28	Stomach	Stomach^55^	20

## Discussion and Conclusion

In this work, we propose a novel heterogeneous network-based method (HNMDRP) to predict the responses of cancer cell lines with multiple drugs based on experimentally IC_50_ values^33^ from the GDSC study^4^. Here, five sub-networks are constructed: (1) cell line similarity network, which is obtained by calculating Pcc values based on cell line gene expression profiles, (2) drug similarity network, which is obtained by calculating Pcc values based on drug chemical structures, (3) target similarity network, which is obtained by merging PPI information and correlational coefficient^34^ based on gene expression profile, (4) cell line-drug association network, which is obtained by log-normalized IC_50_ values from GDSC study, (5) drug-target interaction network, which is obtained by known compound molecular activities. Then a comprehensive heterogeneous network is constructed based on above sub-network. Our main contribution is integrated cell line gene expression profiles, drug chemical structure features, drug-target interactions and PPIs simultaneously. And we demonstrate that known drug-target interactions and PPIs are helpful for improving prediction performance of drug response. The validity of our method is not only supported by its effective in predicting known cell line-drug associations, but also in predicting unknown cell line-drug associations which have dependable literature evidences. Another advantage of our method is the use of correlations among cell lines, drugs, targets. Thus, the huge dimensionality of cell line gene expression profile, drug chemical structure features are not seriously affecting the prediction results.

In addition, as people only concern about whether the specific cancer cell line is sensitive or resistant to a therapy drug, but not what the exact response value is. In this work, we do not learn the exact response value which usually did in previous work^16,35,36^, but studying the binary classification problem (sensitive or resistant)^9^ of the drug response. From the results, we find that for most drugs, our HNMDRP method can obtain the best ROC curves, and the value of AUC is obtained from the corresponding curves. Comprehensive results show that our HNMDRP have achieved slightly better performance than existing state-of-the-art method in predicting drug responses.

Despite our method have achieved encouraging results, it cannot avoid the following limitations which we will extend and improve in future work. Firstly, the construction of cell line similarity network relied only on cell line’s genome-wide gene expression profile data, but not integrating cell line’s somatic mutation, copy number variation^36,37^ which could potentially influence the prediction performance based on our heterogeneous network method^22^. Secondly, the construction of drug similarity network relied on drug’s 1-D and 2-D structural properties which might give sufficient features to represent a drug, but not integrating the 3-D structure features which may play a crucial role for certain drugs. Thirdly, construction of target similarity network relied only on correlational relationship and PPIs^34^, and target sequence information could be analyzed to characterize the similarity among targets. Previous work indicate that sequence information is predictive in drug response^15^. Thus, if effectively incorporate these informative data resources into our model, the predictive performance may be further improved. With increasing data and theoretical support become available over time, we hope our method will have even better prediction results and potentially promote drug discovery process.

## Materials and Methods

In this work, we use GDSC study^4^ as benchmark dataset which is downloaded from website (http://www.cancerrxgene.org/) by Wellcome Trust Sanger Institute. The dataset consist of 1001 cancer cell line and 265 tested drugs, and it also provide gene expression profiles which represent cell line genomic information and a series of continuous IC_50_ values^33^ which represent the drug response measurement. In this work, we use 189 drugs which they have both chemical structure features and drug response data and 962 cell lines which they have both genomic profiles and drug response after data preprocessing. We also extract the interactions between 189 drugs and 243 target genes based on the GDSC dataset. In order to incorporate PPIs into target similarity network, we download totally 4850628 PPIs data from STRING^38^ database and extract 396419 PPI interactions among available 3040 genes which are associated with target genes^39^. We briefly describe the methods of calculating similarities and connections in the following section.

### Cell line similarity network

To construct cell line similarity network, firstly, we separate the baseline gene expression profile of cancer cell line based on genomic data from GDSC. Then we get 962 cell lines with 16383 dimensional gene expression profiles (Fig. 1B left panel). Similar to previously study^16^, the Pearson correlation coefficient^40^ (Pcc) value of each cell line pair is calculated based on their gene expression profiles. Finally, as shown in Fig. 1C, we use a matrix *SIM*_*cc*_ to represent cell line–cell line similarity network which is generated by the Pcc value of all cell line pairs.

### Cell line-drug association network

Initial cell line-drug associations are summarized by the log-normalized IC_50_ values from the GDSC database. We use the threshold provided by Iorio, *et al*.^41^ to classify these continues IC_50_ values into two classes: sensitive or resistant (Fig. 1A). Firstly, the threshold is distinct for each drug, and then the IC_50_ values higher than this threshold are defined as resistant, otherwise are defined as sensitive. Finally, we get overall associations including 17316 sensitive, 129815 resistant and 34687 unknown among 962 cell lines and 189 drugs. As shown in Fig. 1C, we use a matrix *A*_*cd*_ to represent the association network between 962 cell lines and 189 drugs for further analysis.

### Drug similarity network

To construct drug-drug similarity network, firstly, we download drug’s chemical structures from PubChem^42^ (https://www.ncbi.nlm.nih.gov/pccompound) of 189 drugs in which they all have chemical structure features. Then we extract the 1-D and 2-D structure properties (listed in Supplementary Table S1) of 189 drugs using PaDEL software^43^ program with default settings (Fig. 1B middle panel). The 1-D features include compositional molecular properties such as atom count, bond count and molecular weight. And 2-D features consist of various quantitative properties of molecular topology, e.g., Kappa shape indices^44^, Randic^45^ and Wiener indices^46^. Finally, we follow the work of Zhang *et al*.^16^, the Pcc value of each drug pair is calculated based on these features. As shown in Fig. 1C, we use a matrix *SIM*_*dd*_ to represent drug-drug similarity network which is generated by the Pcc value of all drug pairs.

### Drug-target interaction network

In this work, our target information are collected from GDSC^4^ database. First, we extract drug-target interactions among 189 drugs and 243 target genes which also exist in KEGG^47^ drug database. And then, we extract 3040 available genes which are associated with target genes^39^ based on STRING database. Finally, as shown in Fig. 1C, the corresponding matrix *A*_*dt*_ is generated to represent drug-target network among 189 drugs and 3040 genes.

### Target similarity network

To construct target-target similarity network, two different gene-gene relationship matrixes *W*_*ppi*_ and *W*_*corr*_ are generated (Fig. 1B right panel). Firstly, we use 0.4 confidence cut-off value^48,49^ to extract 396419 PPIs between available genes based on STRING database^38^. Similar to the works^50,51^, the confidence score of those PPIs are transformed to matrix *W*_*ppi*_(*i*, *i*). It is normalized as below:

1 $$\overline{Wppi}=Wppi(i,j)/\sqrt{Dppi(i,i)\ast Dppi(j,j)}$$

where ${D}_{ppi}(i,i)$ is the sum of row *i* in ${W}_{ppi}(i,i)$, $\overline{Wppi}(i,j)$ is the normalized matrix which represent the weight of PPIs among available genes. Then we extract gene expression profiles of those available genes based on GDSC database. We follow previous study^39^ and calculate the Pcc value based on gene expression profiles. We use a matrix *W*_*corr*_ to represent the weight of the correlational relationships which is generated by the above calculated Pcc value among available genes^34^. Finally, in order to deal with these two kinds of weighted matrix (*W*_*corr*_ and $\overline{Wppi}$) fairly, we treat them as below^52^:

2 $$SI{M}_{tt}=1-(1-{W}_{corr})\ast (1-\overline{Wppi})$$

As shown in Fig. 1C, we use a matrix *SIM*_*tt*_ denote the target similarity network which is constructed by merging correlational relationship (*W*_*corr*_) and PPI ($\overline{Wppi}$) information.

### HNMDRP

In this work, we propose a novel heterogeneous network-based method (HNMDRP) to efficiently predict cell line-drug associations by making good use of heterogeneous information of cell line gene expression profile, drug chemical structure feature, drug target interaction and PPIs information. The overall workflow of our method is summarized as Fig. 1. Firstly, the Pcc^40^ is a widely used measurement for identifying correlational relationships^34^. And it is defined as:

3 $$Pcc=\frac{{\sum }^{}(X-\bar{X})(Y-\bar{Y})}{\sqrt{{\sum }^{}{(X-\bar{X})}^{2}{\sum }^{}{(Y-\bar{Y})}^{2}}}$$

where X and Y are the column vector of a node’s feature, $\bar{{\rm{X}}}$ and $\bar{{\rm{Y}}}\,\,$are the mean value of each feature vector. Here, we take cell line similarity network as an example. The Pcc value together with the *p-value* (t-test) between this cell line and other cell lines are calculated. We take the procedure of previously study^39^ and use their criteria to choose the cell line pairs with absolute Pcc value which is ranked in top 50% among all cell line pairs and the *p-value* less than 0.01 as correlated, then use such Pcc value as the similarity score. Via this procedure, we can also obtain drug similarity network among 189 drugs. Then we introduce the similarity measure to construct a heterogeneous network model by incorporating complex relationships which include cell line gene expression, drug chemical property, drug-target and PPIs simultaneously. This comprehensive network H(C, D, T, and E) consists of five sub-networks, i.e. cell line-cell line similarity network, drug-drug similarity network, target-target similarity network, cell line-drug association network and drug-target interaction network. These networks are connected by three types of nodes that are defined below: cancer cell line nodes, drug nodes and target gene nodes. Let CC = {*c*_1_, *c*_2_, *c*_3_…*c*_*n*_} denote the *n* cancer cell line nodes, DD = {*d*_1_, *d*_2_, *d*_3_…*d*_*m*_} denote the *m* drug nodes. These two types of node are transformed to similarity matrixes *SIM*_*cc*_ and *SIM*_*dd*_. Here, in each intra-network, the element of *SIM*(*i*, *j*) in row *i* column *j* is the Pcc value between node *i* and node *j*. And *TT* = {${t}_{1},{t}_{2},{t}_{3}\ldots {t}_{l}$} denote the *l* target gene nodes, the element of *SIM*_*tt*_ is obtained by combining PPI and correlational relationships. In addition, we define the weight of the edges between nodes as *CD* = {$c{d}_{ij}$*|i* = *1, 2, 3…n, j* = *1, 2, 3…m*} and *DT* = {*dt*_*ij*_*|i* = *1, 2, 3…m, j* = *1, 2, 3…l*}. The matrix *A*_*cd*_ (*i*, *j*) is the bipartite association network between cell lines and drugs. For instance, the edge (E) $c{d}_{ij}$ is set as 1 if cell line *i* is sensitive to drug *j*, otherwise, resistant or unknown are set to be 0. And the matrix ${A}_{dt}(i,j)$ is also a bipartite graph which is built according to the molecular activity between drugs and target genes. The edge *dt*_*ij*_ is set as 1 if a drug has its corresponding therapeutic target *j*, otherwise is set as 0. Finally, as Fig. 1C shows, a comprehensive heterogeneous network is constructed based on above five similarity and interaction network. Subsequently, an information flow-based algorithm^23^ is performed on this synthetic network as below:

4 $${A}_{cd}^{k+1}=\alpha {A}_{cd}^{k}\times (SI{M}_{dd}\times {A}_{dt}^{k}\times SI{M}_{tt}\times {A}_{dt}^{k\,T})+(1-\alpha ){A}_{cd}^{0}$$

5 $${A}_{dt}^{k+1}=\alpha ({A}_{dt}^{k\,T}\times SI{M}_{cc}\times {A}_{cd}^{k}\times SI{M}_{dd})\times {A}_{dt}^{k}+(1-\alpha ){A}_{dt}^{0}$$

where the matrix ${A}_{cd}^{0}$ and ${A}_{dt}^{0}$ represent the initial cell line-drug associations and drug-target interactions, *SIM*_*cc*_, *SIM*_*dd*_ and *SIM*_*tt*_ are the similarity network among cell line, drug, and target gene, respectively, *α* is the decay factor in the range of 0 to 1. These two equations can be viewed as propagation algorithm across this comprehensive network in the process of iteration^23^. The matrix ${A}_{cd}^{k+1}$ is the final drug response prediction score when the difference between ${A}_{cd}^{k+1}$ and ${A}_{cd}^{k}$ satisfy a sum error with a threshold value of 1e-4^24^. Since different data resources are merged together, proper normalization on matrixes are required to ensure the algorithm can converge^23^. And it is defined as follows:

6 $$Norm({v}_{i},{v}_{j})=\frac{W({v}_{i},{v}_{j})}{\sqrt{{\sum }_{k=1}^{m}\,W({v}_{i},{v}_{k}){\sum }_{k=1}^{n}\,W({v}_{k},{v}_{j})}}$$

where W (v_i_, v_j_) is the matrixes of $(SI{M}_{dd}\times {A}_{dt}^{k}\times SI{M}_{tt}\times {A}_{dt}^{kT})$ or $({A}_{dt}^{kT}\times SI{M}_{cc}\times {A}_{cd}^{k}\times SI{M}_{dd})$ in the process of iteration, *Norm*(*v*_*i*_, *v*_*j*_) is the normalized matrix.

## Electronic supplementary material

Supplementary information

Dataset 1

Dataset 2
