# Supplementary material for: A novel heterogeneous network-based method for drug response prediction in cancer cell lines
Source: Sci Rep. 2018 Feb 20;8:3355. doi: 10.1038/s41598-018-21622-4 (PMC5820329; doi:10.1038/s41598-018-21622-4)
Supplement: Supplementary file 1 — Supplementary information [file 41598_2018_21622_MOESM1_ESM.pdf]

A novel heterogeneous network-based method for drug response prediction in cancer cell lines

Fei Zhang[1,+], Minghui Wang [1,2,+,*], Jianghong Yang[2] , Jianing Xi[2] , and Ao Li[1,2]

[1]School of Information Science and Technology, University of Science and Technology of China, Hefei AH230027, China

[2]Centers for Biomedical Engineering, University of Science and Technology of China, Hefei AH230027, China

*Correspondence and requests for materials should be addressed to M.W. (email: mhwang@ustc.edu.cn)

[+] These authors contributed equally to this work

Supplementary Information

Supplementary Figure S1. The ROC curve of drug GSK2126458.

Supplementary Figure S2. The ROC curve of drug NVP-BHG712.

Supplementary Figure S3. The ROC curve of drug TPCA-1.

Supplementary Figure S4. The predictive performance when removing different information (RDSI - only remove drug structure information, RPPI - only remove PPI information, RGCI - only remove gene-gene correlation information, RTI - only remove target information).

Supplementary Figure S5. The AUC values of three major tissue types when only using these tissue to train our model.

Supplementary Table S1. The 189 drugs and its corresponding chemical structure features.

Supplementary Table S2. The drug name and ID number of 189 drugs.

Supplementary Table S3. The AUC values of all drugs.

Supplementary Table S3. The AUC and AUPR values of all cell line types of tissues.

Figure

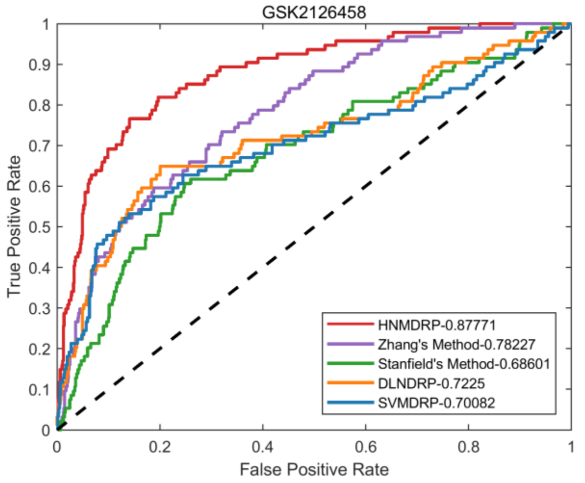

Figure S1. The ROC curve of drug GSK2126458 among five methods.

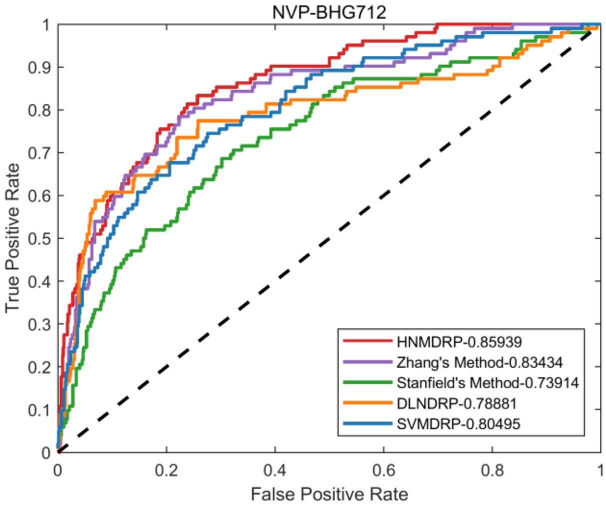

Figure S2. The ROC curve of drug NVP-BHG712 among five methods.

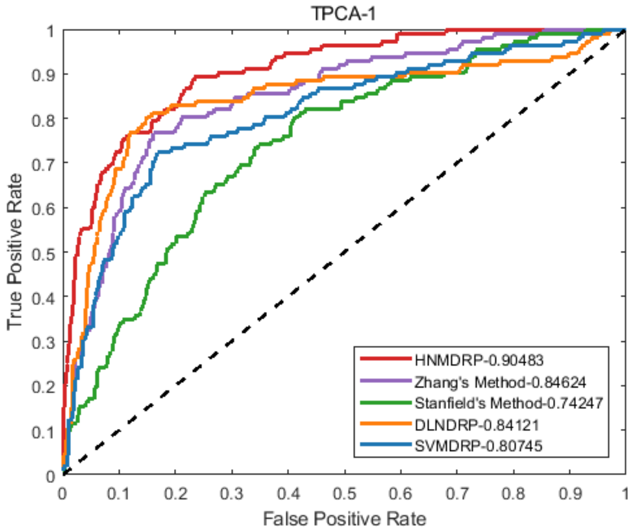

Figure S3. The ROC curve of drug TPCA-1 among five methods.

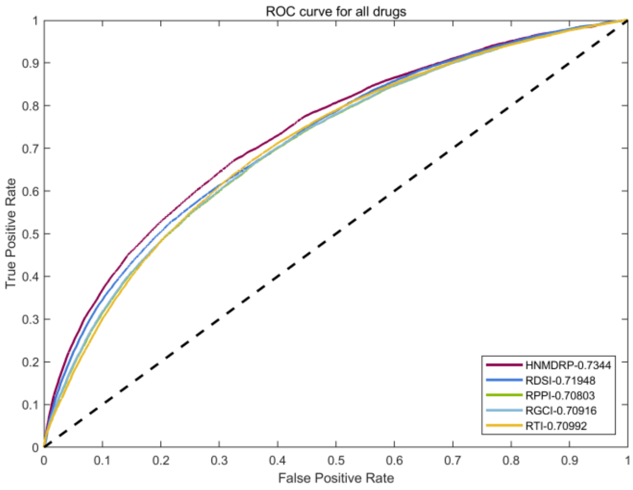

Figure S4. The ROC curve for all drugs when only using each information in our HNMDRP method.

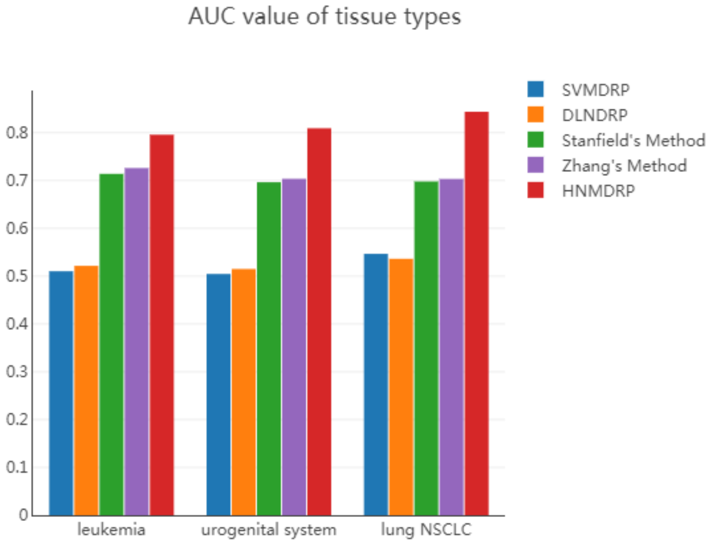

Figure S5. The AUC values of three major tissue types when only using these tissue to train our model.

Table

Table S3. Results of HNMDRP, DLNDRP and SVMDRP on drug response predictions using leave-one-out cross validation (LOOCV). For most drugs, HNMDRP achieves the best AUC values than other four methods.

	AUC value of five methods				
Drug Name	HNMDRP	Zhang's Method	Stanfield's Method	DLNDRP	SVMDRP
SNX-2112	0.9380	0.9079	0.7523	0.8896	0.8938
CAY10603	0.9341	0.9103	0.7733	0.8708	0.8692
TG101348	0.9283	0.8560	0.7721	0.8295	0.8199
CP466722	0.9143	0.8669	0.7727	0.8581	0.5955
TPCA-1	0.9048	0.8462	0.7425	0.8412	0.8074
BIX02189	0.9007	0.8596	0.7882	0.8360	0.8486
JW-7-24-1	0.9001	0.8387	0.7475	0.7921	0.8295
AT-7519	0.8974	0.8805	0.7601	0.8642	0.7724
Belinostat	0.8917	0.8194	0.7156	0.8343	0.8302
TAK-715	0.8893	0.8328	0.7274	0.7377	0.7849
ZSTK474	0.8870	0.8239	0.7076	0.8237	0.8170
PI-103	0.8856	0.8365	0.6837	0.8016	0.8223
UNC0638	0.8846	0.8832	0.7212	0.8148	0.7548
OSI-930	0.8781	0.8187	0.7353	0.8157	0.6349
CUDC-101	0.8780	0.8409	0.7176	0.8359	0.7899
GSK2126458	0.8777	0.7823	0.6860	0.7225	0.7008
Sunitinib	0.8753	0.7566	0.6662	0.6598	0.6763
OSI-027	0.8752	0.8041	0.7271	0.7929	0.7404
PIK-93	0.8749	0.8104	0.7499	0.7944	0.7936
T0901317	0.8715	0.8351	0.7507	0.8325	0.8145
BX-795	0.8714	0.7397	0.6062	0.7406	0.5042
BX-912	0.8710	0.7873	0.6962	0.7908	0.7781
XL-880	0.8668	0.7444	0.6384	0.7396	0.7145
IPA-3	0.8653	0.8198	0.6600	0.8251	0.8020
NG-25	0.8646	0.7946	0.7065	0.7870	0.8380
I-BET-762	0.8633	0.7983	0.7529	0.8078	0.7874
GW843682X	0.8595	0.7398	0.5860	0.6337	0.3932
NVP-BHG712	0.8594	0.8343	0.7391	0.7888	0.8050
AR-42	0.8579	0.8475	0.7269	0.8402	0.6757
YM201636	0.8560	0.8351	0.7189	0.8070	0.8081
VNLG/124	0.8559	0.8173	0.7250	0.8230	0.4289
Etoposide	0.8556	0.7810	0.6270	0.7306	0.5325
SB-715992	0.8487	0.8040	0.6769	0.7523	0.7228
AV-951	0.8473	0.8272	0.6944	0.7844	0.8000
CAL-101	0.8469	0.7602	0.6710	0.7538	0.7473
BAY 61-3606	0.8454	0.7366	0.6251	0.6880	0.6916
FMK	0.8408	0.7378	0.6975	0.7381	0.6799

Zibotentan	0.8401	0.8121	0.7308	0.8079	0.7489
S-Trityl-L-cysteine	0.8397	0.7804	0.5913	0.7456	0.7610
GSK1070916	0.8392	0.7767	0.7037	0.8151	0.6273
BMS-345541	0.8331	0.7240	0.6715	0.7232	0.7360
Crizotinib	0.8318	0.7662	0.5967	0.7051	0.7602
Masitinib	0.8301	0.7262	0.6674	0.7183	0.6489
LAQ824	0.8293	0.6588	0.6287	0.6976	0.5512
XL-184	0.8288	0.7384	0.6740	0.6986	0.6903
ABT-869	0.8274	0.7256	0.6358	0.6411	0.7312
Vorinostat	0.8235	0.7817	0.6366	0.7536	0.7784
ZM-447439	0.8217	0.7632	0.6711	0.7843	0.7604
Tubastatin A	0.8171	0.7134	0.6727	0.7118	0.5883
GSK429286A	0.8169	0.7913	0.7192	0.7654	0.7428
EKB-569	0.8168	0.7783	0.5738	0.6411	0.6263
EX-527	0.8166	0.7290	0.6194	0.7315	0.6996
VX-680	0.8130	0.6926	0.5681	0.6782	0.6672
TL-2-105	0.8123	0.7321	0.7249	0.7475	0.4812
CMK	0.8110	0.7845	0.5873	0.7154	0.5405
CGP-082996	0.8103	0.7598	0.6241	0.6620	0.7313
Sorafenib	0.8082	0.7387	0.6045	0.7165	0.6633
Ruxolitinib	0.8073	0.7715	0.6603	0.7810	0.5122
AZD8055	0.8048	0.7659	0.6957	0.7519	0.4448
AC220	0.8032	0.7478	0.6575	0.7164	0.7176
BI-2536	0.8032	0.7393	0.3860	0.6303	0.7562
AP-24534	0.8022	0.8075	0.6324	0.6966	0.6172
Vismodegib	0.7964	0.6816	0.5868	0.7059	0.4722
BMS-509744	0.7960	0.7541	0.6354	0.5423	0.6970
Camptothecin	0.7943	0.8192	0.6862	0.7691	0.6485
CEP-701	0.7942	0.7390	0.5300	0.7107	0.7202
XMD11-85h	0.7922	0.5832	0.4190	0.6714	0.6037
GDC0941	0.7903	0.7140	0.5554	0.6847	0.6871
Olaparib	0.7880	0.6934	0.6264	0.6822	0.4906
FR-180204	0.7865	0.7237	0.6296	0.7527	0.7139
BMS-708163	0.7854	0.7175	0.6814	0.6647	0.7250
Y-39983	0.7854	0.6859	0.6342	0.7393	0.6581
Phenformin	0.7831	0.7598	0.6046	0.6795	0.6619
AICAR	0.7811	0.7631	0.6400	0.7478	0.7203
Cyclopamine	0.7796	0.7524	0.5729	0.7387	0.7156
PD-0332991	0.7788	0.8087	0.6403	0.7037	0.6775
HG-6-64-1	0.7785	0.7359	0.6421	0.7640	0.5708
Axitinib	0.7783	0.7728	0.5446	0.7235	0.7131
VX-702	0.7778	0.7333	0.6545	0.7303	0.4975
AZD7762	0.7769	0.7623	0.6047	0.7461	0.7037

QS11	0.7722	0.6207	0.5465	0.6356	0.6439
Bicalutamide	0.7716	0.7003	0.5772	0.6953	0.5568
KU-55933	0.7705	0.6838	0.5540	0.7259	0.5911
PAC-1	0.7693	0.7923	0.6634	0.7502	0.7398
PFI-1	0.7642	0.6817	0.5670	0.6830	0.6754
Parthenolide	0.7610	0.6829	0.6432	0.6575	0.5653
A-443654	0.7594	0.6734	0.6167	0.5462	0.4456
AUY922	0.7591	0.6754	0.6396	0.5978	0.5262
Embelin	0.7583	0.7395	0.6067	0.6937	0.4241
Nilotinib	0.7581	0.6955	0.5894	0.6828	0.4703
Z-LLNle-CHO	0.7578	0.7044	0.4881	0.6568	0.6509
OSU-03012	0.7526	0.6404	0.5246	0.6244	0.5487
CGP-60474	0.7517	0.7997	0.6460	0.5373	0.7697
MS-275	0.7489	0.7681	0.5663	0.6671	0.6302
Salubrinal	0.7462	0.6609	0.4915	0.7164	0.7074
GSK690693	0.7433	0.6982	0.6757	0.7426	0.6800
CH5424802	0.7420	0.7228	0.6577	0.7299	0.6556
DMOG	0.7409	0.7532	0.5791	0.6785	0.5346
Obatoclax Mesylate	0.7404	0.7253	0.6035	0.5956	0.5496
Tipifarnib	0.7394	0.5820	0.5576	0.5766	0.4762
ATRA	0.7338	0.7135	0.6513	0.6812	0.6933
Bosutinib	0.7274	0.7838	0.6472	0.7295	0.6281
AG-014699	0.7245	0.7074	0.5952	0.6741	0.6494
CP724714	0.7244	0.5966	0.5480	0.6256	0.5943
NVP-TAE684	0.7208	0.7161	0.5293	0.5800	0.6921
JQ1	0.7199	0.6644	0.5464	0.6131	0.5543
AS605240	0.7196	0.7137	0.6894	0.6649	0.5776
Roscovitine	0.7191	0.6169	0.4913	0.6236	0.4910
SN-38	0.7190	0.7715	0.5699	0.7054	0.5173
MK-2206	0.7189	0.6822	0.6093	0.5953	0.5710
VX-11e	0.7165	0.6812	0.7053	0.6754	0.5423
LY317615	0.7130	0.6665	0.5890	0.6751	0.5616
Rapamycin	0.7128	0.5958	0.6546	0.5865	0.6635
XMD8-92	0.7119	0.5886	0.5265	0.6452	0.4141
GNF-2	0.7095	0.6269	0.4096	0.5840	0.6378
GW-2580	0.7090	0.6774	0.6183	0.5926	0.4946
SL 0101-1	0.7074	0.5943	0.5187	0.6293	0.4733
Nutlin-3a	0.7072	0.6904	0.8085	0.6636	0.6530
SB590885	0.7062	0.6938	0.6672	0.6904	0.5309
Temsirolimus	0.7061	0.6998	0.6205	0.6770	0.5959
CCT018159	0.7053	0.5859	0.5188	0.6100	0.5835
Bexarotene	0.7044	0.6271	0.4985	0.5796	0.4864
JNJ-26854165	0.6993	0.6762	0.5357	0.6545	0.5439

Dabrafenib	0.6991	0.7806	0.7151	0.6490	0.5943
BMN-673	0.6963	0.7193	0.6032	0.6882	0.5949
ABT-888	0.6914	0.6853	0.6181	0.6612	0.5551
BMS-536924	0.6903	0.6804	0.5917	0.5042	0.7349
Midostaurin	0.6871	0.6987	0.6126	0.6810	0.5167
PHA-665752	0.6862	0.6410	0.5625	0.5699	0.6321
AZD-0530	0.6854	0.6863	0.5846	0.5433	0.7478
BMS-754807	0.6847	0.6484	0.6269	0.4597	0.5249
PF-562271	0.6841	0.6413	0.5702	0.6024	0.4808
Tamoxifen	0.6817	0.6325	0.5825	0.6507	0.7476
AZ628	0.6805	0.8072	0.7175	0.7254	0.8676
SGC0946	0.6791	0.6670	0.6561	0.7355	0.5738
GSK269962A	0.6753	0.6879	0.5396	0.6008	0.4401
LFM-A13	0.6708	0.6090	0.5086	0.5753	0.5371
Pazopanib	0.6681	0.6826	0.6268	0.5882	0.5423
Thapsigargin	0.6680	0.6696	0.5016	0.5786	0.5871
681640	0.6655	0.6091	0.5905	0.5617	0.6151
PLX4720	0.6649	0.7454	0.6989	0.5861	0.5802
A-770041	0.6645	0.6334	0.4931	0.5693	0.7088
PF-4708671	0.6633	0.5929	0.5397	0.5958	0.6139
Imatinib	0.6630	0.6454	0.6168	0.6442	0.6536
CCT007093	0.6604	0.6137	0.5251	0.5899	0.5987
SB-505124	0.6553	0.6038	0.5527	0.5661	0.5779
AZD6482	0.6528	0.6159	0.5500	0.5748	0.5466
FK866	0.6515	0.6461	0.7274	0.6184	0.6135
(5Z)-7-Oxozeaenol	0.6485	0.7139	0.6975	0.6674	0.5547
NVP-BEZ235	0.6458	0.6347	0.4843	0.5813	0.5115
ABT-263	0.6456	0.7595	0.7108	0.7342	0.6927
AS601245	0.6455	0.5644	0.5859	0.4678	0.4685
OSI-906	0.6423	0.6933	0.6196	0.5484	0.4663
CI-1040	0.6415	0.7890	0.7362	0.5139	0.5593
MP470	0.6402	0.5958	0.5743	0.5830	0.6053
SB 216763	0.6396	0.6512	0.4881	0.5765	0.5455
PD-173074	0.6365	0.6166	0.5719	0.5885	0.6189
RDEA119	0.6325	0.8102	0.7344	0.4200	0.3952
Elesclomol	0.6290	0.6391	0.5046	0.5298	0.5678
GSK-1904529A	0.6251	0.5183	0.6013	0.5074	0.5022
CHIR-99021	0.6223	0.6477	0.6088	0.5572	0.5993
NSC-207895	0.6222	0.6153	0.5788	0.5429	0.5700
MLN4924	0.6220	0.6779	0.5236	0.5627	0.5400
WH-4-023	0.6187	0.6973	0.6144	0.5222	0.7842
AKT inhibitor VIII	0.6184	0.5653	0.5366	0.4447	0.4938
JNK Inhibitor VIII	0.6170	0.6009	0.4621	0.5045	0.5805

EHT 1864	0.6167	0.6170	0.5144	0.6442	0.5981
Dasatinib	0.6102	0.6983	0.6361	0.5091	0.8160
Bryostatin 1	0.6086	0.5988	0.5015	0.5602	0.5105
IOX2	0.6058	0.6084	0.5641	0.5523	0.5326
RO-3306	0.6053	0.6513	0.5837	0.5740	0.6118
FTI-277	0.6051	0.6780	0.5603	0.5768	0.5257
TW 37	0.5879	0.7059	0.6271	0.5526	0.5365
AMG-706	0.5775	0.6302	0.5440	0.6039	0.5324
NSC-87877	0.5743	0.5835	0.5133	0.5114	0.5586
17-AAG	0.5688	0.6204	0.6716	0.4591	0.4475
Gefitinib	0.5641	0.7697	0.7201	0.7828	0.3796
AZD6244	0.5616	0.7103	0.6783	0.4708	0.4841
rTRAIL	0.5580	0.6181	0.5450	0.6138	0.5265
PD-0325901	0.5558	0.7627	0.7135	0.4957	0.3896
Trametinib	0.5506	0.7647	0.7023	0.7484	0.3914
GW 441756	0.5501	0.5118	0.5521	0.4929	0.5036
Erlotinib	0.5455	0.6193	0.6190	0.4147	0.7234
TGX221	0.5421	0.6645	0.5481	0.5674	0.4778
XAV 939	0.5167	0.6882	0.6081	0.5002	0.5126
Afatinib	0.4964	0.8113	0.7231	0.8177	0.4070
Lapatinib	0.4863	0.6210	0.5962	0.3442	0.3716
Cetuximab	0.4639	0.7078	0.5826	0.4100	0.6233
YM155	0.4423	0.5740	0.4913	0.5156	0.3882

Table S4. Results of HNMDRP, DLNDRP and SVMDRP on drug response predictions of different tissue types using leave-one-out cross validation (LOOCV). For every cancer tissue type, HNMDRP achieves the consistent performance with highest AUC and AUPR values.

	AUC value of different tissues			AUPR value of different tissues		
	HNMDRP	DLNDRP	SVMDRP	HNMDRP	DLNDRP	SVMDRP
aero_dig_tract	0.6359	0.4648	0.5489	0.1458	0.0881	0.1184
bone	0.6873	0.5065	0.5574	0.2597	0.1502	0.1672
breast	0.6674	0.4829	0.5210	0.1261	0.0650	0.0721
digestive_system	0.6705	0.5561	0.5379	0.1799	0.1141	0.1096
kidney	0.6369	0.4878	0.5529	0.1427	0.0917	0.1523
large_intestine	0.6536	0.5204	0.5179	0.1277	0.0966	0.0827
leukemia	0.6831	0.5185	0.6140	0.4577	0.2897	0.3796
lung	0.7011	0.4758	0.5134	0.1239	0.0607	0.0733
lung_NSCLC	0.6769	0.5462	0.5065	0.1373	0.0853	0.0745
lung_SCLC	0.6953	0.6022	0.5605	0.1719	0.0997	0.0902
lymphoma	0.7287	0.5527	0.5937	0.4037	0.2376	0.2651
myeloma	0.6538	0.5065	0.5777	0.2658	0.1723	0.1992
nervous_system	0.6623	0.5308	0.5677	0.1342	0.0861	0.1127
neuroblastoma	0.6352	0.5969	0.5211	0.1867	0.1641	0.1122
pancreas	0.7151	0.4720	0.5308	0.1731	0.0734	0.0793
skin	0.6041	0.4663	0.5006	0.1559	0.0968	0.1109

soft_tissue	0.6221	0.5028	0.5579	0.1736	0.1193	0.1688
thyroid	0.6709	0.6144	0.5669	0.1809	0.1527	0.1293
urogenital_system	0.6761	0.5149	0.5397	0.1830	0.0944	0.1064
